# Efficacy of various extracting solvents on phytochemical composition, and biological properties of *Mentha longifolia* L. leaf extracts

**DOI:** 10.1038/s41598-023-45030-5

**Published:** 2023-10-21

**Authors:** Meryem Tourabi, Amira Metouekel, Asmae E. L. ghouizi, Mohamed Jeddi, Ghizlane Nouioura, Hassan Laaroussi, Md. Eram Hosen, Kawtar Fikri Benbrahim, Mohammed Bourhia, Ahmad Mohammad Salamatullah, Hiba-Allah Nafidi, Gezahign Fentahun Wondmie, Badiaa Lyoussi, Elhoussine Derwich

**Affiliations:** 1 Laboratory of Natural Substances, Pharmacology, Environment, Modeling, Health & Quality of Life, Faculty of Sciences, Sidi Mohamed Ben Abdellah University, Fez, Morocco; 2 Euromed Research Center, Euromed Faculty of Pharmacy, Euromed University of Fes (UEMF) Route de Meknes, 30000 Fez, Morocco; 3 The Higher Institute of Nursing Professions and Health Techniques (ISPITS), Fez, Morocco; 4 Laboratory of microbial biotechnology and bioactive molecules, science and technology faculty sidi Mohamed ben Abdellah University, Imouzzer, Road, Fez, Morocco; 5 Department of Genetic Engineering and Biotechnology, University of Rajshahi, Rajshahi, 6205 Bangladesh; 6 Department of Chemistry and Biochemistry, Faculty of Medicine and Pharmacy, Ibn Zohr University, 70000 Laayoune, Morocco; 7 Department of Food Science and Nutrition, College of Food and Agricultural Sciences, King Saud University, 11 P.O. Box 2460, 11451 Riyadh, Saudi Arabia; 8 Department of Food Science, Faculty of Agricultural and Food Sciences, Laval University, 2325, Quebec City, QC G1V 0A6 Canada; 9 Department of Biology, Bahir Dar University, P.O. Box 79, Bahir Dar, Ethiopia; 10 Unity of GC/MS and GC-FID, City of Innovation, Sidi Mohamed Ben Abdellah University, Fez, Morocco

**Keywords:** Biotechnology, Drug discovery, Plant sciences

## Abstract

The current work attempts to explore the influence of three extraction solvents on phytochemical composition, content of polyphenols, antioxidant potential, and antibacterial capacity of hydroethanolic, acetonic, and aqueous extracts from Moroccan *Mentha longifolia* leaves. To achieve this goal, the chemical composition was identified using an HPLC–DAD examination. The contents of polyphenols were assessed, while the total antioxidant capacity (TAC), the DPPH test, and the reducing power test (RP) were utilized to determine antioxidant capacity. To assess the antibacterial activity, the microdilution technique was carried out to calculate the minimum inhibitory (MIC) and minimum bactericidal concentrations (MBC) of extracts against four nosocomial bacteria (*Bacillus cereus*, *Pseudomonas aeruginosa****,**** Escherichia coli****,*** S*taphylococcus aureus*). Additionally, the antibacterial and antioxidant activities of all tested extracts were examined in silico against the proteins NADPH oxidase and *Bacillus cereus* phospholipase C. Study reveals that *M. longifolia* extracts contain high phenolic and flavonoids. Additionally, the hydroethanolic extract contained the highest amounts of phenolic and flavonoid content, with values of 23.52 ± 0.14 mg Gallic acid equivalent/g dry weight and 17.62 ± 0.36 mg Quercetin Equivalent/g dry weight, respectively compared to the other two extracts. The same extract showed the best antioxidant capacity (IC_50_ = 39 µg/mL ± 0.00), and the higher RP (EC_50_ of 0.261 ± 0.00 mg/mL), compared to the acetonic and aqueous extract regarding these tests. Furthermore, the hydroethanolic and acetonic extracts expressed the highest TAC (74.40 ± 1.34, and 52.40 ± 0.20 mg EAA/g DW respectively), compared with the aqueous extract. Regarding antibacterial activity, the MIC value ranges between 1.17 and 12.50 mg/mL. The in-silico results showed that the antibacterial activity of all extracts is principally attributed to kaempferol and ferulic acid, while antioxidant capacity is attributed to ferulic acid.

## Introduction

Aromatic medicinal plants (AMPs) are commonly employed in both folk medicine and gastronomy, primarily due to their great concentration of polyphenols, which offer several other advantageous properties including antioxidant and antimicrobial activity^1^. Species of the *Mentha* genus are rich in antioxidants^2^. Because of their antioxidant capacity, these compounds have various therapeutic qualities, including antibacterial, anti-inflammatory, anti-diabetic, and cardioprotective effects, with low toxicity and high efficiency^3^. *Mentha longifolia,* also known as "naana touil" in Morocco, is a traditional medicinal plant that grows naturally and spontaneously in the Moroccan mountains. It is used for its therapeutic and medicinal characteristics*. M. longifolia*, like other mint species, is frequently used in the manufacture of herbal teas and spices. This plant is widely recognized in conventional medicine for its potential as antimicrobial, antioxidant, analgesic, antiemetic, antimutagenic, and anti-cancer. In addition, it was reported that *M. longifolia* is used in folk medicine to treat inflammatory and menstrual disorders^4,5^. Nowadays, bacterial resistance to antibiotic drugs has been recognized as a problem since the beginning of the antibiotic era. Nevertheless, it is only in the last twenty years that the development of dangerous and resistant bacteria has become an alarming regularity^6^. Antibiotic resistance is globally spreading as a result of the misuse or overuse of antibiotics, as well as insufficient infection control and precautions. A growing list of microbial infections such as tuberculosis, blood poisoning, pneumonia, gonorrhea, and foodborne illnesses is becoming more difficult and nearly impossible to treat^7^. Because of the problem of antibiotic resistance, researchers are currently focusing on bioactive components derived from common herbal species used in phytotherapy, which may generate a new potential source of antibacterial activity^8^. In addition, medicinal plants are an enormous source of bioactive molecules, reduce the risk of several diseases like oxidative stress, neurodegenerative disorders, cerebral vascular accidents, cancer, and coronaropathies, to some allergies or affections, repairing, toning, sedative, revitalizing or immunomodulating effects^9,10^.

The current work attempts to examine the impact of various extraction solvents on the phytochemical composition, antioxidant potential, and in vitro antibacterial activity of Moroccan *Mentha longifolia* leaf extracts against resistant nosocomial bacteria. Furthermore, the *in-silico* study was also evaluated.

## Materials and methods

### Plant material

The plant was collected in May 2021 in the town of Ifran (Moroccan Middle Atlas), and it was identified by Professor Amina BARI, a botanist at the Sidi Mohamed Ben Abdellah University. The voucher specimen has been deposited at the faculty herbarium number: 001MLAV202162. Afterward, the collected plant was dried in the dark for two weeks, and then the aerial parts were removed and powdered using a professional herbs grinder. The obtained powder was packed in a glass flask and stored in an obscure and heat-protected locale until extraction.

### Preparation of extracts

In the current study, various solvents with different polarities were used to prepare the various extracts, and the extraction process was carried out, with minor modifications in agreement with El Ghouizi et al.,^11^. Shortly, 10 mL of each selected solvent (ethyl acetate, chloroform, hexane, hydroethanolic 70% v/v, acetone, and distilled water) were added to 1 g of plant powder, and the mixture was macerated and sustained under continuous agitation at room temperature for one week. After a previously performed test, which examined a variety of solvents, these conditions showed the most promising results (data not shown). Whatman N°1 filter was used to remove impurities from the extracts and thereafter condensed using a rotary vacuum evaporator at 40 °C. The resulting crude extracts were collected and kept at −20 °C before use.

### Determination of dry matter content

Using a rotary evaporator under vacuum (BUCHI Rotavapor R­200) at 40 °C, the extracts were concentrated to obtain crude extract. Furthermore, the yield of each extract was calculated and expressed as a percentage and determined using the procedure below (1).

1 $${\mathrm{Y\,extract\, \%}} = [({\mathrm{Weight \, of  \, dry  \, extract}} ({\mathrm{g}}))/({\mathrm{Weight \, of  \, dry  \, pant}} ({\mathrm{g}}))\times 100]$$

Y extract is extraction yield expressed in % (w/w).

### HPLC–DAD analysis

Organic extracts of *Mentha longifolia* leaf extracts were characterized utilizing reverse-phase high-performance liquid chromatography with a dad array detector (HPLC–DAD). For the analysis, a MOS-1 HYPERSIL 250 4.6 mm SS Exsil ODS 5 m analytical column with a Thermo Scientific HPLC system was used. The separation was achieved in gradient mode using two solvents, A (water) and C (acetonitrile), and the elution gradient was as follows: 80% A, 20% C for 1 min, 60%A, 40 percent C for 2.5 min, and 80% A, 20% C for 4 min. The injection volume was 5 µL, and the flow rate was kept at 1 mL/min. The standard of polyphenolic components: gallic acid, ferulic acid, kaempferol, quercetin vanillic acid, and naringenin were determined by correlating their retention time and UV spectra^12^.

### Evaluation of the antioxidant potential

#### Free radical scavenging activity (DPPH)

1,1-diphenyl-2-picrylhydrazyl (DPPH) is a violet-colored free radical reduced in the presence of antiradical compounds. The DPPH radical scavenging activity of our samples was conducted using the procedure reported by Brand Williams et al.^13^, with minor changes. Briefly, 825 µL of DPPH ethanolic solution (65 μM), was added to 50 µL of each extract and standard solution. Absorbance readings were obtained at 517 nm after 60 min of incubation in the dark. The IC_50_ values were determined to represent the 50% inhibition of DPPH radicals using the following formula (2).

2 $$\% inhibition=[(Abscontrol-Abssample/Abscontrol)\times 100]$$

BHT was chosen as a standard and the IC_50_ value was calculated from the percentage of the inhibition curve.

#### Reducing power assay

To determine the reducing power of our samples, we employed the method outlined by Shams Moattar et al.^14^. Briefly, 50 μL volume of plant extracts was added to 250 μL of phosphate buffer (0.2 M, pH 6.6) and 250 µL of potassium ferricyanide (1%). 250 µL of acetic trichloride acid (10%) was added to the mixture after 20 min of incubation in a water bath at 50° C, and the mixture was centrifuged at 3000 rpm for 10 min. 250 µL of the supernatant was then added to 250 µL of distilled water and 60 μL of ferric chloride (0.1%). The extract concentration that provides 0.5 of absorbance (EC_50_) was calculated from the graph of absorbance, and ascorbic acid was employed as a positive control. The absorbance was measured at 700 nm.

#### Total antioxidant power test

Total antioxidant capacity (TAC) was conducted according to Mašković et al.^15^. The test for phosphomolybdate is based on the extract's transformation of Mo (VI) to Mo (V) and the formation of a green Mo (V) phosphate complex at an acidic PH. Shortly, 50 µL of sample extracts were combined with 1 mL of phosphomolybdenum reagent (0.6 M sulfuric acid, 28 mM sodium phosphate, and 4 mM ammonium molybdate). After 90 min in a 95 °C boiling water bath, absorbance readings were taken at 695 nm against a blank. Results were expressed as milligrams of ascorbic acid equivalent per gram of plant dry weight (mg AAE /g DW), and the standard curve was established using ascorbic acid as a standard.

### Estimation of total phenolic content

The total phenolic content (TPC) of our samples was estimated according to El Ghouizi et al.^11^ utilizing the Folin Ciocalteu approach. 50 µL of each sample was combined with 500 µL of the Folin solution (0.2 N). 400 µL of a sodium carbonate solution (75 g/L) was added after incubating for 5 min. The mixture was incubated for two hours in the obscure, and the absorbance was read at 760 nm. The standard curve was created using the gallic acid solution, and the TPC was calculated as the quantity of gallic acid equivalent in milligrams per gram of plant dry weight (mg GAE/g DW).

### Estimation of flavonoid content

The acetic trichloride (AlCl3) test was carried out to assess the flavonoid content in the various samples^16^. 500 µL of AlCl3 reagent (10%) were added to 500 µL of plant extracts and standard solution. After one hour of incubation in the darkness, absorbances were measured at 420 nm. The flavonoid concentration was represented as milligrams of quercetin equivalent per gram of plant dry weight (mg QE /g DW), and the standard curve was established using quercetin as a standard.

### Evaluation of the antibacterial activity

#### Tested Microorganisms

Four strains were selected to investigate the antibacterial properties of our extracts, including two Gram-negative microbes, *Escherichia coli* (25,922), *Pseudomonas aeruginosa* (27,853), and two Gram-positive bacteria, *Staphylococcus aureus* (29,213 (ATCC))***,*** and *Bacillus cereus* (6633). The selected strains were kept on nutritional agar (NG) and the sensitivity test was performed on Mueller–Hinton agar (MHB). On the selected bacterial strains, the different extracts were examined, firstly to calculate the minimum inhibitory concentration (MIC) using a liquid medium at 0.2% and secondly in the solid state to indicate the minimum bactericide concentration (MBC).

#### Preparation of bacterial suspension

The various bacteria were cultured in 9 mL of MHB for 18–24 h at 37 °C. Besides fresh culture, the bacterial suspension was prepared using a sterile platinum loop where one or two colonies were collected and placed in a tube containing sterile physiological water (0.9%) and then the bacterial suspension was homogenized using a vortex. The optical density was calibrated at 0.5 Mc Farland at 625 nm. The spectroscopic density of the inoculum was updated to a concentration of 10^7^ to 10^8^ CFU/ ml^17^.

#### Determination of the lowest inhibitory concentration (MIC)

The Lowest Inhibitory Concentration (MIC) represents the lower concentration capable of inhibiting any visible growth after an incubation time of 18 to 24 h. The MIC of our different extracts (Hydroethanolic, Acetone, and water) was determined by microdilution technique in microplates under sterile conditions^18^. The examination of the minimum inhibitory concentration (MIC) was carried out by dilution of different extracts in dimethyl sulfoxide DMSO (2%)^19^. The first line wells were dispersed with 50 µl (50 mg/ml) of each extract for inoculation, 50 µl of MH was therefore added, and a series of dilutions were carried out. The MIC was calculated by adding 10 µL of Resazurin (7-Hydroxy-3H-phenoxazin-3-one 10-oxide) after the microplate had been incubated for one day at 37 °C (0.015 percent). Color is a strong indicator of the existence of bioactive microorganisms.

#### Determination of the minimal bactericidal concentration (MBC)

The MBC is the concentration of antibacterial that leaves no more than 0.01% of surviving germs^20^. After the MIC determination, samples are taken from every well free of bacterial growth placed on MH agar, and incubated at 37 °C. After a 24-h incubation time, the MBC is measured^21^.

### In silico assessment

#### Ligand preparation

Phytochemicals present in extracts from the leaves of *Mentha longifolia* were identified using HPLC. These phytochemicals were selected for a docking study and their 3D structures in sdf format were obtained from PubChem^22^. The ligands were then prepared using Avogadro software (version: 1.2.0), where their structures were constructed and refined using the mmff94 force field. This process involved eliminating undesired groups, including hydrogen atoms, and optimizing the energy and geometry of the ligand structures^23^.

#### Protein preparation

Due to the notable antibacterial activity of the extract against *Bacillus cereus*, the docking study aimed to explore interactions with specific target proteins. These proteins included Phospholipase C (PDB ID: 2huc) from *Bacillus cereus*^24^, and NADPH Oxidase from *Lactobacillus sanfranciscensis* (PDB ID: 2cdu)^25^. The 3D structures of these proteins, obtained from the Protein Data Bank (PDB), were in x-ray diffraction format. Employing Discovery Studio (version: 4.5) and Swiss pdb viewing software (version: 4.1), extraneous molecules like heteroatoms, water molecules, and non-target components were eliminated from the protein structures before initiating the docking process. To enhance the stability, the protein structures underwent energy minimization using the GROMACS96 force field. Following this, ligands were generated, and the modified protein structures were employed for conducting docking studies in pdb format.

#### Molecular docking

To explore the potential inhibitors derived from plant extracts for their antibacterial effects against *Bacillus cereus*, as well as their potential as antioxidants, a molecular docking study was conducted. The PyRx software tool (version: 0.8) was employed for this purpose^26^. The ligands were initially inputted using Open Babel and subsequently converted into PDBQT format. Additionally, the target protein was introduced into PyRx, with the protein being converted into macromolecules in PDBQT format. For the specific proteins, the center and grid box dimensions were set for optimal docking. For the protein with PDB ID 2huc, the coordinates were X: 88.3723 Å, Y: 68.9215 Å, Z: 14.0256 Å, and the grid box size was X: 43.3734 Å, Y: 47.8133 Å, Z: 57.55 Å. Similarly, for the protein with PDB ID 2cdu, the coordinates were X: 10.7221 Å, Y: 5.0086 Å, Z: 25.7258 Å, and the grid box size was X: 65.8874 Å, Y: 71.0272 Å, Z: 101.0575 Å. Docking calculations were executed using the PyRx program, while the analysis of binding interactions was carried out using Discovery Studio. The docking investigation involved utilizing the lowest binding scores, and the energy values were estimated in kcal/mole.

## Statistical analysis

The different assays performed in this work were conducted in triplicate, and the findings have been expressed as mean ± SD. GraphPad Prism 9 software was used for the statistical analyses, along with one-way ANOVA and Tukey's multiple comparisons. One-way ANOVA and Tukey's multiple comparisons were used in the statistical studies, which were carried out using GraphPad Prism 9 software. Principal component analysis (PCA) was carried out with the use of the STATISTICA program, and Pearson's correlation coefficient (r) was utilized to determine relations between the examined variables.

## Results and discussion

### Solvent screening

In phytochemical research, the most critical step in the extraction of phenolic and other bioactive constituents from vegetables, fruits, and plants is solvent selection. In summary, different factors, such as solvent polarity, temperature, and time, influence the extraction efficiency of phenolic components, and their effects can be independent or coupled^27^. In this work, results of the preliminary screening showed that three solvents (ethyl acetate, chloroform, and hexane) showed the most minimal effect in the antiradical scavenging activity, the reducing power, and the antioxidant activity. These three solvent extracts showed also a lower amount of total phenolic compounds. According to our preliminary results, the antioxidant power of *M. longifolia* was greatly impacted by the polarity of the extraction solvent which was confirmed by Aazza et al.^28^. The total phenolic and flavonoid content of the different assessed extracts varied between 7.130 ± 0.786 to 23.524 ± 0.139 mg GAE/g DW, and from 3.757 ± 0.255 to 17.622 ± 0.359 mg QE/g DW respectively. It should be noted that the hydroethanolic extract exhibited greater TPC and TFC levels. Considering the preliminary screening performed based on solvent polarity, we decided to select the stronger extracts (hydroethanolic, acetonic, and water) to perform the phenolic composition and antibacterial activities, and their results will be represented and discussed in the remaining sections of this paper.

### Extraction yields

Choosing the best extraction solvent mixture depending on material properties is crucial to attaining high yields. As solvents for extraction, ethanol 70%, acetone, and water were used in Table 1. The findings indicate that the high extraction yield was recorded during extraction with 70% EtOH with a rate of 10.5 ± 0.074%, followed by water with a rate of 2.25 ± 0.016%, however, acetone solvent achieved the lowest yield with a rate of 1.9 ± 0.013%. According to researchers, the polarity of the solvent has a substantial effect on extract yield^29^. The extract yield in polar solvents (water and EtOH 70%) was reported to be greater than in nonpolar solvents (acetone). Brahmi and coworkers reported that hydro-alcoholic solvents had the maximum extraction yield. Whereas ethanol (at 75%) provided the best extraction rates for Algerian *Mentha spicata* (20.02%), acetone gave the lowest rate (2.6%)^30^.

**Table 1:** Extractive yields of *M. longifolia* leaf extracts (% dry weight).

	Water	EtOH 70%	Acetone
*M.longifolia* Leaves	2.25 ± 0.016	10.5 ± 0.74	± 0.013

### HPLC–DAD examination of phenolic compounds

**Figure 1:**
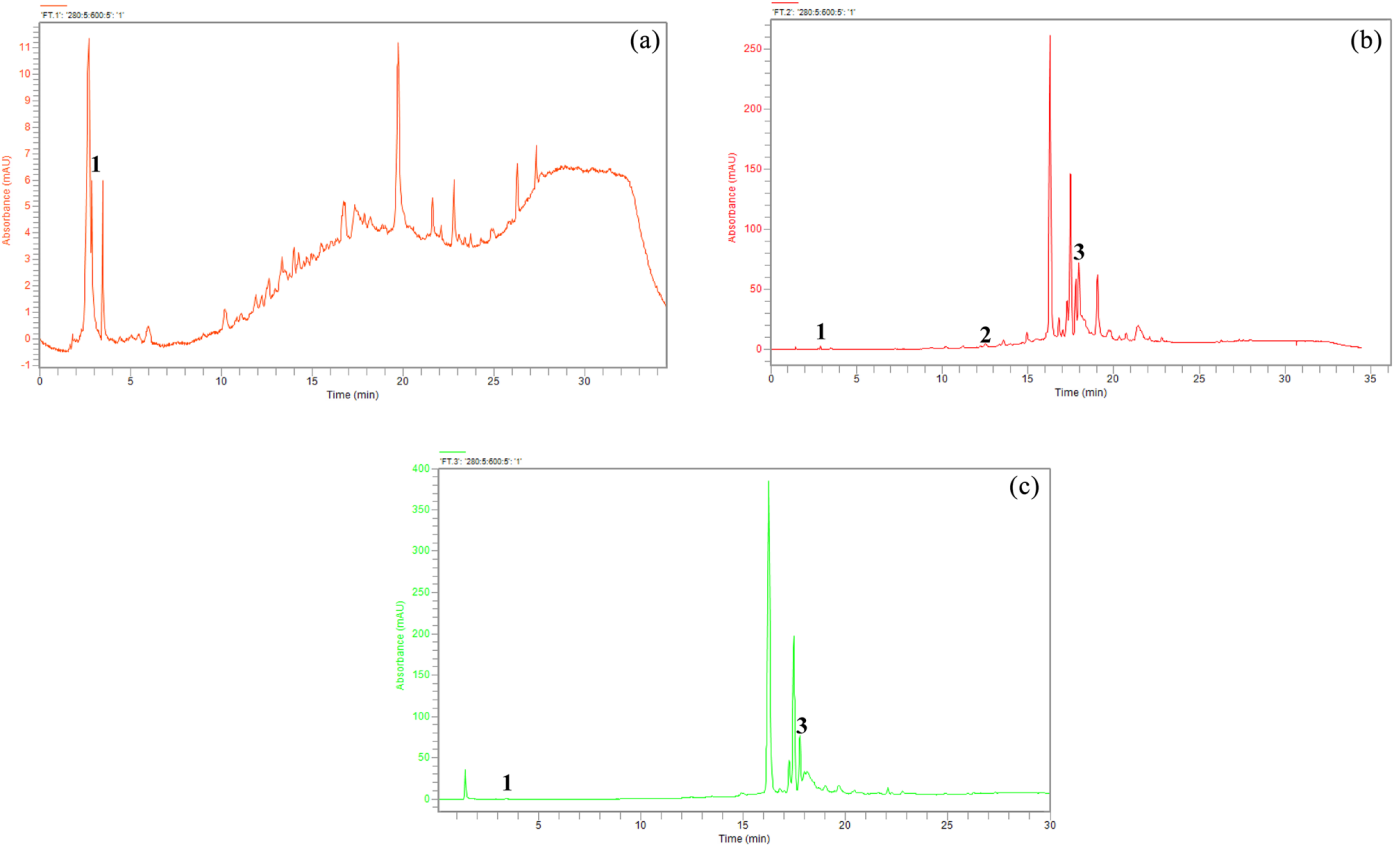
HPLC–DAD chromatogram of *M. longifolia* leaves extracts (**a**) aqueous (**b**) ETOH70% (**c**) acetone.

HPLC–DAD chromatograms of the aqueous, hydroethanolic, and acetonic extracts are shown in Table 2 and Figs. 1 (a, b, and c), respectively. The chemical profiles of the three extracts vary considerably, indicating the distinct efficacy of each extraction method. The aqueous extract contains mainly compounds with a retention time of around 19.732 min, as shown by the highest peak in Fig. 1. These are rather polar molecules, which is quite normal in an aqueous extract. This extract contains gallic acid, known for its antioxidant properties. Gallic acid has been associated with a variety of health advantages, including potential antioxidant effects^4^. The hydroethanolic extract reveals a diverse range of compounds, with significant peaks around 16.281 min (non-identified) and 17.975 min (kaempferol). A range of moderately polar to apolar molecules, demonstrating the efficiency of the mixed ethanol/water solvent. Kaempferol, present in hydroethanolic extract, is a flavonoid known for its antioxidant and anti-inflammatory potential. The acetone extract shows a distinct chemical profile, with significant peaks at around 16.258 min (non-identified) and 17.988 min (kaempferol). This is an extract containing molecules that are rather medium in polarity. The presence of chlorobenzoic acid and kaempferol in the acetone extract shows that the acetone extract may contain compounds with antimicrobial, and antioxidant properties^31^, similar to those observed in the hydroethanolic extract. Our data are in line with studies^4,32^, of other plant extracts. Our findings highlight the distinct chemical profiles of the three extracts, suggesting the effectiveness of each extraction method. These results confirm the value of *Mentha longifolia* extracts as potential sources of bioactive compounds with diversified therapeutic potential, mainly antioxidant and possibly anti-bacterial activity.

**Table 2:** HPLC–DAD chromatographic study of individual phenolic composition identified in the various extracts of *Mentha longifolia* leaves.

Peak	Standards	RT (min)	Area %		
			Water	EtOH 70%	Acetone
1	Gallic acid	2.886	6.22	0.14	0.01
2	Ferulic acid	12.236	ND	0.14	ND
3	Kaempferol	17.975	ND	6.05	2.80

ND: Non detected.

### Phenolic and flavonoid content of the different Mentha longifolia leaf extracts

Phenolic compounds are the most common type of phytochemical found in a wide range of plant-based products, including phenolic acids (hydroxycinnamic acids, and hydroxybenzoic), polyphenols (condensed tannins, and hydrolyzable), flavonoids (rutin, naringenin, quercetin, apigenin, kaempferol)^33^, because of its importance in the pharmaceutical, and food industries, these compounds have been extensively researched^34^. In this context, *M. longifolia* (Horsemint) is considered a potential source of bioactive compounds as well as micronutrients, which can be explored as a promising alternative for the formulation of nutraceutical products^35^. As previously confirmed by our results, the phenolic content strongly depends on the selected extraction solvent.

**Figure 2:**
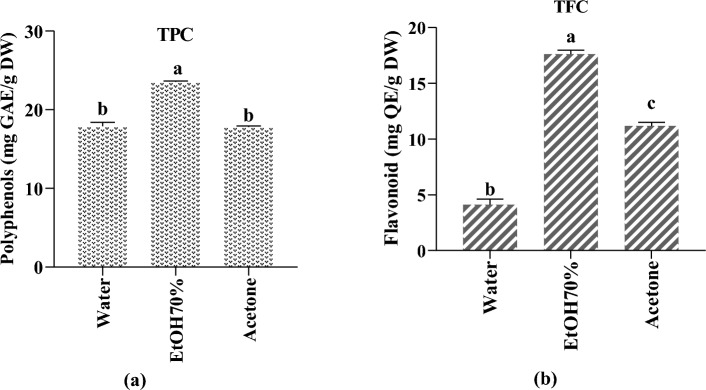
(**a**) Total phenolic content (TPC) of *M. longifolia* extracts. (**b**) Total flavonoid content (TFC) of *M. longifolia* extracts. Tukey's multiple range test showed that results with the same letter in the same test are not statistically different (p < 0.05).

Figure 2 (a) demonstrates the data of the total phenolic content of the tested *M. longifolia* extracts, and found that aqueous extracts had the lowest value (17.90 0.49 mg GAE/g DW), whereas acetonic extracts had the greatest value (23.52 0.14 mg GAE/g DW). This study suggests that hydroethanolic was the most efficient extraction solvent for extracting polyphenols, followed by acetone and water, suggesting that polar solvents extract more phenolic content than apolar solvents. Our results are higher than those reported by Patonay et al.,^36^ and Motamed and coworkers^37^. In contrast, Ertaş*et al*. found that the phenolic content of methanol, acetone, and petroleum ether extracts ranged from 217.10 ± 1.82 to 225.65 ± 3.42 µg GAE/mg and were higher than our extracts^38^.

Regarding flavonoids content results are summarized in Fig. 2 (b) and suggested that the hydroethanolic extract showed the highest amount of flavonoids (17.622 ± 0.359 mg QE/g DW), followed by acetone extract (11.174 ± 0.330 mg QE/g DW), whereas aqueous extract showed the lowest amounts with (4.114 ± 0.497 mg QE/g DW), these findings were inferior to those found by Ertaş et al.^38^ and Hajlaoui et al.^39^.

### Antioxidant activity of the different Mentha longifolia leaf extracts

**Figure 3:**
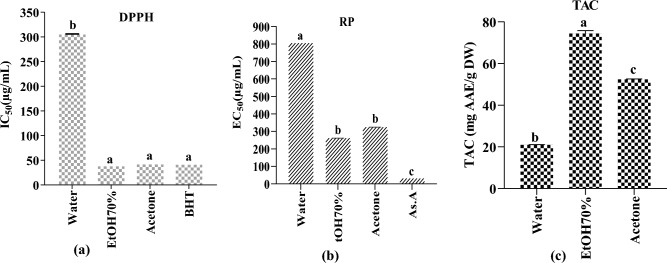
(**a**) Radical scavenging activity of *M. longifolia* leaf extracts (DPPH assay), (**b**); reducing power of *M. longifolia extracts*; (**c**) Total antioxidant activity (TAC) of *M. longifolia* extracts. Tukey's multiple range test showed that results with the same letter in the same test are not statistically different (p < 0.05).

Results of the antiradical property (DPPH), reducing power, (PR), and the total antioxidant capacity (TAC) of the selected *Mentha longifolia* leaf extracts were summarized in Fig. 3 and showed a significant variation between the different extracts. Our findings showed that hydroethanolic extract exhibited the highest total antioxidant capacity (74.40 ± 1.34 mg AAE/g) followed by acetone extract (52.40 ± 0.20 mg AAE/g), and aqueous extract which showed the lowest value (20.98 ± 0.08 mg AAE/g) (Fig. 3 (c)). In the same way, the aqueous extract presented the lowest radical scavenging inhibition with the highest IC_50_ = 306 ± 0.1 µg/mL for the DPPH test and the highest EC_50_ = 0.80 ± 0.03 mg/mL for the reducing power (RP) compared to standards. However, the hydroethanolic extract showed higher free radical scavenging activity (DPPH) and reducing power (RP) (IC_50_ = 39.00 ± 0.00 µg/mL and EC_50_ = 0.261 ± 0.00 mg/mL respectively) followed by the acetone extract (IC_50_ = 43.00 ± 0.00 µg/mL and EC_50_ = 0.324 ± 0.00 mg/mL respectively) showing no statistical significance with BHT and ascorbic acid used as standards (Fig. 3 (a, b)). These outcomes were stronger than those reported by Bahadoi et al.^5^ for the Iranian *M. longifolia* ethanolic and aqueous extracts in which they reported DPPH IC_50_ values ranged between 195.96 ± 0.94, and 162.08 ± 3.90 mg TEs/g extract, and RP EC_50_ values ranged between 239.87 ± 3.95, and 346.20 ± 0.17 mg TEs/g sample. The findings of this study showed that hydroethanolic solvent is the most effective for extraction and it had the highest levels of phenolic and flavonoid content, which were closely related to the strong antioxidant activities (DPPH, TAC, and RP) that were noticed. It was followed by acetone and water. Our findings are consistent with those previously published, which reported that high-polarity solvents are extensively used to extract antioxidant compounds such as hydroethanolic, water, acetone, and methanol. It is well known that ethanol, methanol, and water are polar solvents commonly used to extract polar molecules like phenolic and flavonoid components^40^.

Previous studies have been conducted to identify the phenolic and flavonoid profiles of *M. longifolia* and revealed a variety of natural antioxidant molecules such as phenolic acids (gallic acid, vanillic acid, rosmarinic acid, caffeic acid, syringic acid, ρ-coumaric acid, o-coumaric acid, ferulic acid, trans-cinnamic acid, and chlorogenic acid) and flavonoids (naringenin, rutin, quercetin, apigenin, kaempferol)^36,41,42^. Iranian authors^5^ stated that rosmarinic acid and cinnamic acid were principally identified in their *M. longifolia* areal part extracts. Furthermore, flavone glycosides such as apigenin and luteolin were the major flavonoids detected in the Moroccan *M. longifolia* areal part extracts^43^. The findings of the present work suggest that the strong antioxidant activity of *M. longifolia* extracts is mostly attributed to its phenolic profile^5,44^.

### Evaluation of the antibacterial activity

The outcomes of the antibacterial effect of *M. longifolia* leaf extracts are represented in Tables 3 and 4. In this study, a total of 4 nosocomial pathogenic microbial strains (2 Gram-positive and 2 Gram-negative) were used to investigate the antibiotic potential of our samples. Results showed that all extracts revealed strong bacterial growth inhibition against all tested pathogenic bacteria. In particular, acetone extract showed the highest inhibitory effect against *Bacillus cereus* with a MIC = 1.17 ± 0.05 mg/mL, and MBC = 1.50 ± 0.05 mg/mL, and *Staphylococcus aureus*, (MIC = 2.34 ± 1.10 mg/mL, and MBC = 6.25 ± 0.43 mg/mL). Additionally, acetone extract exhibited higher activity against *Escherichia coli* with a MIC = 6.25 ± 0.00 mg/mL. It is crucial to record that acetone extract has exhibited a powerful antibacterial effect against Gram-positive bacteria, more than Gram-negative bacteria except *Pseudomonas aeruginosa*. Acetone plant extract tends to be more effective in terms of antibacterial activity; indeed, Felhi et al.^45^ reported that the acetone extract of fruit bark and seeds of *E. elaterium* signaled the highest activity against *Staphylococcus aureus* and *Bacillus subtilis*. However, Zhang et al.,^46^ have demonstrated in their research that the ethanolic extract of *Mentha arvensis* exhibited strong antibacterial activity against *acinetobacter baumannii*. Another study on methanolic extract of the aerial parts of *M. longifolia ssp*. had no antibacterial effect^4^. Preeti e*t al.*^47^ evaluated the antibacterial efficacy of several *Mentha piperita* leaf solvent extracts against pathogenic bacteria notably *Pseudomonas aeruginosa, Staphylococcus aureus*, *Klebsiella pneumonia*, *Escherichia coli*, *Proteus vulgaris,* and results revealed that aqueous and ethyl acetate extracts exhibited the strongest antibacterial activity.

**Table 3:** Minimum Inhibitory Concentration (MIC) of *M. longifolia* extracts in gram-negative and gram-positive bacteria (mg/mL).

Extracts	Gram-negative Bacteria		Gram-positive Bacteria	
	*P. aeruginosa*	*E. coli*	*S. aureus*	*B. cereus*
Etoh70%	6.25 ± 0.00^a^	12.50 ± 0.00 ^a^	4.68 ± 0.21^a^	3.12 ± 0.00^a^
Acetone	12.50 ± 0.00^b^	6.25 ± 0.00^b^	2.34 ± 0.11^b^	1.17 ± 0.05^ab^
Water	9.37 ± 0.41^a^	12.50 ± 0.00^a^	4.68 ± 0.21^a^	4.61 ± 0.76^a^

Tukey’s multiple range test showed that results with the same letter in the same test are not statistically different (p < 0.05).

**Table 4:** Minimal bactericidal concentration (MBC) *M. longifolia* extracts in gram-negative and gram-positive bacteria (mg/mL).

Extracts	Gram-negative Bacteria		Game-positive Bacteria	
	*P. aeruginosa*	*E. coli*	*S. aureus*	*B. cereus*
Etoh70%	25 ± 1.28	25 ± 3.78	25 ± 2.26 ^a^	1.56 ± 0.07
Acetone	–	–	6.25 ± 0.43 ^b^	1.50 ± 0.05
Water	–	–	–	–

Tukey’s multiple range test showed that results with the same alphabet in a similar test are not statistically distinct (p < 0.05).

In our current study, we noticed that Gram-positive strains were more responsive to our extracts compared to Gram-negative strains. The reason for the lower sensitivity of Gram-negative bacteria could be attributed to the complexity of their double membrane, which includes a cell envelope made up of a lipoprotein and a lipopolysaccharide layer (LPS). Unlike Gram-positive bacteria's single membrane, this structure works as a biological barrier to antibacterial drugs^48^. The demonstrated antibacterial activity of our *Mentha longifolia* extracts can be explained by its rich phytochemical profile, which includes caftaric acid, rosmarinic acid, cryptochlorogenic acid, ρ-coumaric acid, m-coumaric acid, chlorogenic acid, caffeic acid, gallic acid, luteolin, apigenin, quercetin, rutin, coumarins, and isocoumarins^49,50^. Indeed, previous studies have reported greater antimicrobial potency for caffeic, cryptochlorogenic, as well as chlorogenic acids^49,51,52^. Other phenolic compounds found in various species of the genus *Mentha* such as ellagic acid, ferulic acid, gallocatechin, epigallocatechin gallate, and catechins were reported to exhibit antibacterial or bacteriostatic effects against multiple bacterial strains including *Escherichia coli*, *Staphylococcus aureus*, *Bacillus aureus*, *Bacillus pumilis*, *Bacillus subtilis*, and *Pseudomonas aeruginosa.* This effect is associated with their capability to penetrate the bacterial wall and reach the bacterial cytoplasm^52^.

As stated previously, several solvent combinations have been applied to effectively extract phenolic components from plant material. Water, ethanol, methanol, acetone, and their aqueous mixes are the most commonly used solvents^53^. The significant antibacterial impact found in our investigation can be attributed to acetone's ability to extract antimicrobial compounds due to its power to dissolve hydrophilic and lipophilic components^54^. Furthermore, acetone is the most effective extractor of plant material since it can extract molecules with a wide range of polarities and has low toxicity in biological assays, making it a highly valuable extraction solvent^55^. Interestingly, acetone was shown to be the most effective solvent for extracting polyphenols, such as hydroxycinnamic acids (ferulic acid, sinapic acid, caffeic acid, ρ-coumaric acid, chlorogenic acid, luteolin, quercetin, vanillic acid, and catechin), and hydroxybenzoic acids (gallic acid, and protocatechuic acid)^56^. These molecules have been mentioned to have a powerful antibacterial effect through several mechanisms, notably membrane instability, membrane hyper-permeabilization, hyper-acidification, enzyme inhibition, and the generation of reactive quinones^57^. According to Campos et al.,^58^ these acids might also inhibit the synthesis of nucleic acids by Gram-negative and Gram-positive bacteria. Due to their propenoid side chain, hydroxycinnamic acids are slightly more polar than corresponding hydroxybenzoic acids, which may assist their transit through the cell membrane increasing their toxic effect into the bacterial cell^59,60^. In the same context, gallic and ferulic acids have been shown to cause bacterial cell death by altering bacterial hydrophobicity (interacting with the surface of Gram-negative and Gram-positive bacteria), potentially acidifying their cytoplasm by increasing K + release and inducing protein denaturation, which can change the cytoplasmic membrane permeability, induce intracellular material release, and cause membrane damage.^60^. Moreover caffeic acid, and ρ-coumaric acid impact cell membrane structure through inflexibility and alterations in the phospholipid chains' stability^61^.

### In silico assessment

#### Molecular docking against Phospholipase C Bacillus cereus

**Figure 4:**
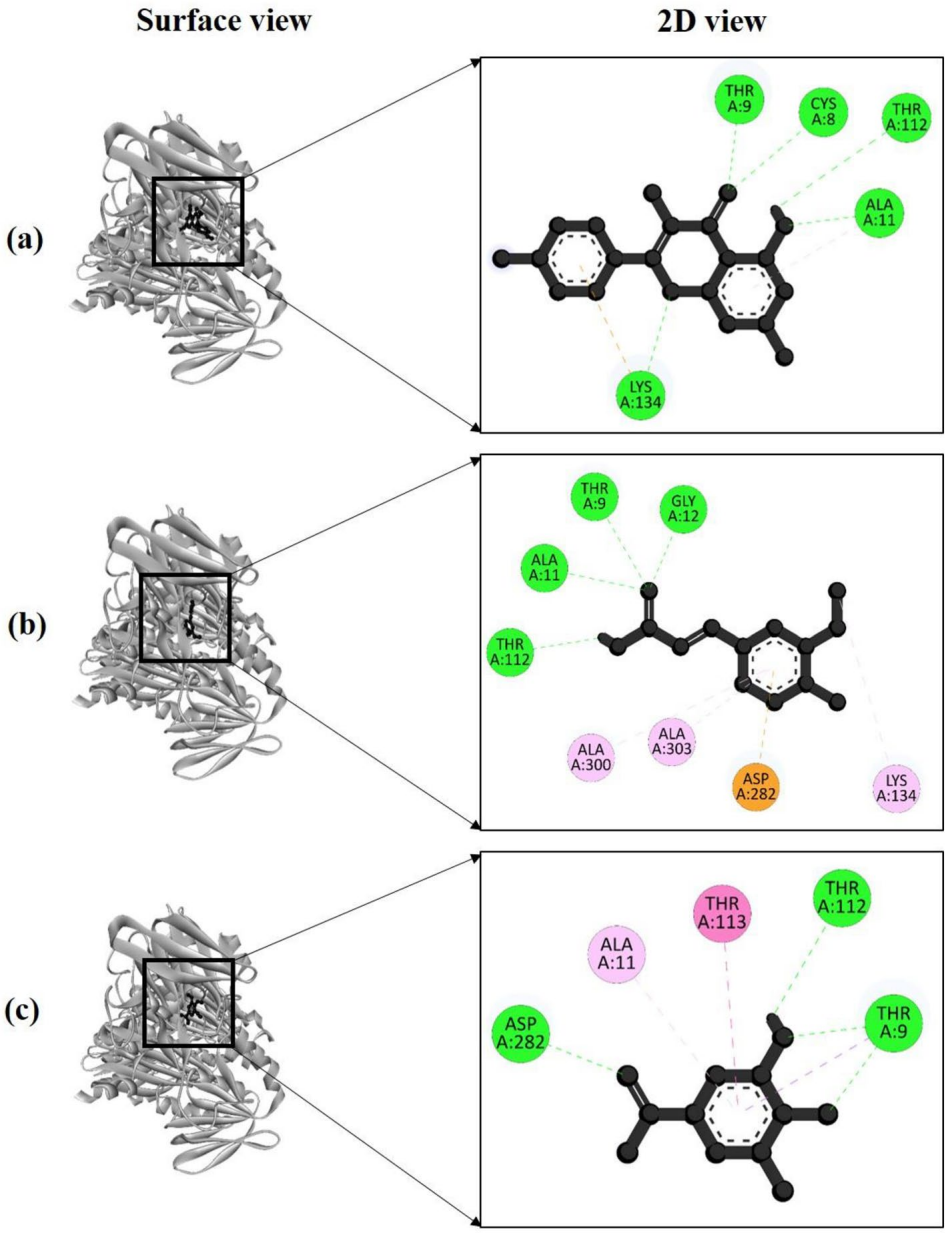
The molecular docking study by Phospholipase C (PDB ID: 2huc) of *Bacillus cereus* and the active compounds from extract; surface and 2 D view of compounds (**a**) Kaempferol, (**b**) Ferulic acid and (**c**) Gallic acid respectively.

Three compounds identified from *M. longifolia* leaf extracts through HPLC were subjected to molecular docking studies. The findings indicated that all three compounds bind to the same region within the active site (Fig. 4). Among these compounds, Kaempferol displayed the highest binding energy at −9.67 kcal/mol, followed by Ferulic acid at −9.5 kcal/mol and Gallic acid at −8.75 kcal/mol (Table 5).

**Table 5:** Binding energy, interacting amino acid residues, bond types, and their distance between Phospholipase C (PDB ID: 2huc) of *Bacillus cereus* and the three docked compounds.

Complex	Binding energy (kcal/mole)	Amino acid residues	Bond types	Distance (Å)
2cdu + Kaempferol	–9.67	Cys8	H	2.83
		Thr9	H	2.00
		Ala11	H	2.30
		Thr112	H	2.95
		Lys134	H	3.00
2cdu + Ferulic acid	–9.5	Thr9	H	2.43
		Ala11	H	2.40
		Gly12	H	2.17
		Thr112	H	2.27
		Lys134	A	4.97
		Ala300	A	5.35
		Ala303	A	4.76
		Asp282	PIA	3.45
2cdu + Gallic acid	–8.75	Thr9	H	2.27
		Thr112	H	2.78
		Asp282	H	2.12
		Ala11	PA	5.12
		Thr113	AS	4.75

The interaction analysis between Phospholipase C (PDB ID: 2huc) and the top-performing compound kaempferol revealed the formation of five hydrogen bonds at specific amino acid residues: Cys8 (2.83 Å), Thr9 (2.00 Å), Ala11 (2.30 Å), Thr112 (2.95 Å), and Lys134 (3.00 Å) (Fig. 4a). In the case of 2cdu and Ferulic acid, there were four hydrogen bonds formed with Thr9 (2.43 Å), Ala11 (2.40 Å), Gly12 (2.17 Å), and Thr112 (2.27 Å) (Fig. 4b). Notably, both Kaempferol and Ferulic acid shared a hydrogen bond at the same position, suggesting a similar interaction pattern with the target protein.

Additionally, the third compound, Gallic acid, exhibited interactions with the target protein 2huc, forming three hydrogen bonds at Thr9 (2.27 Å), Thr112 (2.78 Å), and Asp282 (2.12 Å) (Fig. 4c).

#### Molecular docking against NADPH oxidase

**Figure 5:**
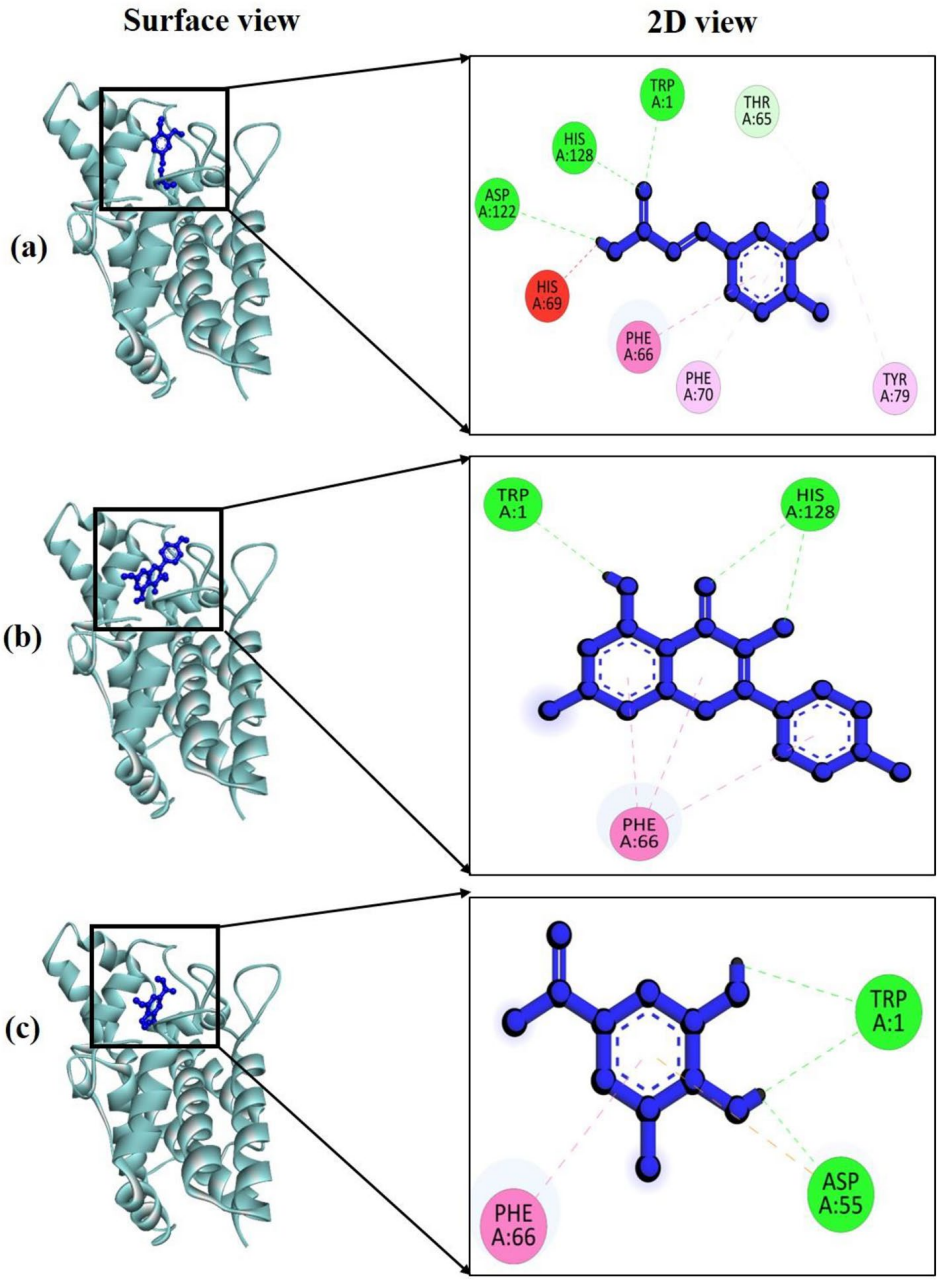
The molecular docking study by targeting NADPH oxidase (PDB ID: 2cdu) and the HPLC detected active compounds from extract; surface and 2 D view of compounds (**a**) Ferulic acid, (**b**) Kaempferol and (**c**) Gallic acid.

In terms of binding affinity to the target enzyme NADPH oxidase, Ferulic acid exhibited the strongest interaction with a binding energy of −8.4 kcal/mol, followed by Kaempferol and Gallic acid with binding energies of −8.0 and −7.9 kcal/mol, respectively (Table 6). Ferulic acid formed interactions with NADPH oxidase (PDB ID: 2cdu) through four hydrogen bonds, which significantly contributed to its binding stability. These hydrogen bonds were observed with specific amino acid residues: Trp1 (2.05 Å), Asp122 (2.72 Å), His128 (2.83 Å), and Thr65 (3.63 Å) (Fig. 5a).

**Table 6:** Binding energy, interacting amino acid residues, bond types, and their distance between NADPH oxidase (PDB ID: 2cdu) and the three docked compounds.

Complex	Binding energy (kcal/mole)	Amino acid residues	Bond types	Distance (Å)
2huc + Ferulic acid	−8.4	Trp1	H	2.05
		Asp122	H	2.72
		His128	H	2.83
		Thr65	H	3.63
		Phe70	A	4.86
		Tyr79	A	4.56
		Phe66	A	3.96
		His69	PIA	1.42
2huc + Kaempferol	−8	Trp1	H	2.28
		His228	H	2.67
		Phe66	PPS	4.11
2huc + Gallic acid	−7.9	Trp1	H	2.46
		Asp55	H	1.85
		Phe66	PPS	4.43

On the other hand, both Kaempferol and Gallic acid exhibited an equivalent number of hydrogen bonds when interacting with the target protein. Specifically, they formed hydrogen bonds with Trp1 (at 2.28 Å) and His228 (at 2.67 Å) for Kaempferol (Fig. 5b), and with Trp1 (at 2.46 Å) and Asp55 (at 1.85 Å) for Gallic acid (Fig. 5c), respectively.

## Statistical analysis

### Correlation test

The correlation test is well-known for showing any causal relationships between the various factors studied in the research. The results are summarized in Table 7 and exhibited a strong excellent correlation between phenolic, and flavonoid contents (r = 0.845), and total antioxidant capacity (TAC) (r = 0.802 and 0.997 respectively Adversely, phenolic content and the antioxidant activity measured by DPPH and RP showed a considerable negative correlation (r = −0.498. and −0.571 respectively), and between the antioxidant tests themselves, DPPH/TAC (r = −0.917), and TAC/ RP (r = −0.948). Our results demonstrated also a strong positive correlation between the polyphenolic content and the antibacterial activity of the studied *M. longifolia* extracts against all microbiological strains. Our data are consistent with those previously published which reported a positive relationship between the antioxidant capacity, phenolic content, and the antibacterial efficiency^62,63^.

**Table 7:** Pearson correlation coefficients.

	Polyphenols	Flavonoids	TAC	DPPH	RP
Polyphenols	–	0.845	0.802	−0.498	−0.571
Flavonoids	0.845	–	0.997*	−0.885	−0.922
TAC	0.802	0.997*	−	−0.917	−0.948
DPPH	−0.498	−0.885	−0.917	−	0.996*
RP	−0.571	−0.922	−0.948	0.996*	−
*P. aeruginosa*	−0.874	−0.477	−0.410	0.013	0.099
*E. coli*	0.513	−0.026	−0.101	0.489	0.411
*S. aureus*	0.511	−0.029	−0.104	0.491	0.414
*B. cereus*	0.078	−0.468	−0.534	0.826	0.774

Statistical significance. *p < 0.05, **p < 0.01, ***p < 0.001.

### Principal component analysis

**Figure 6:**
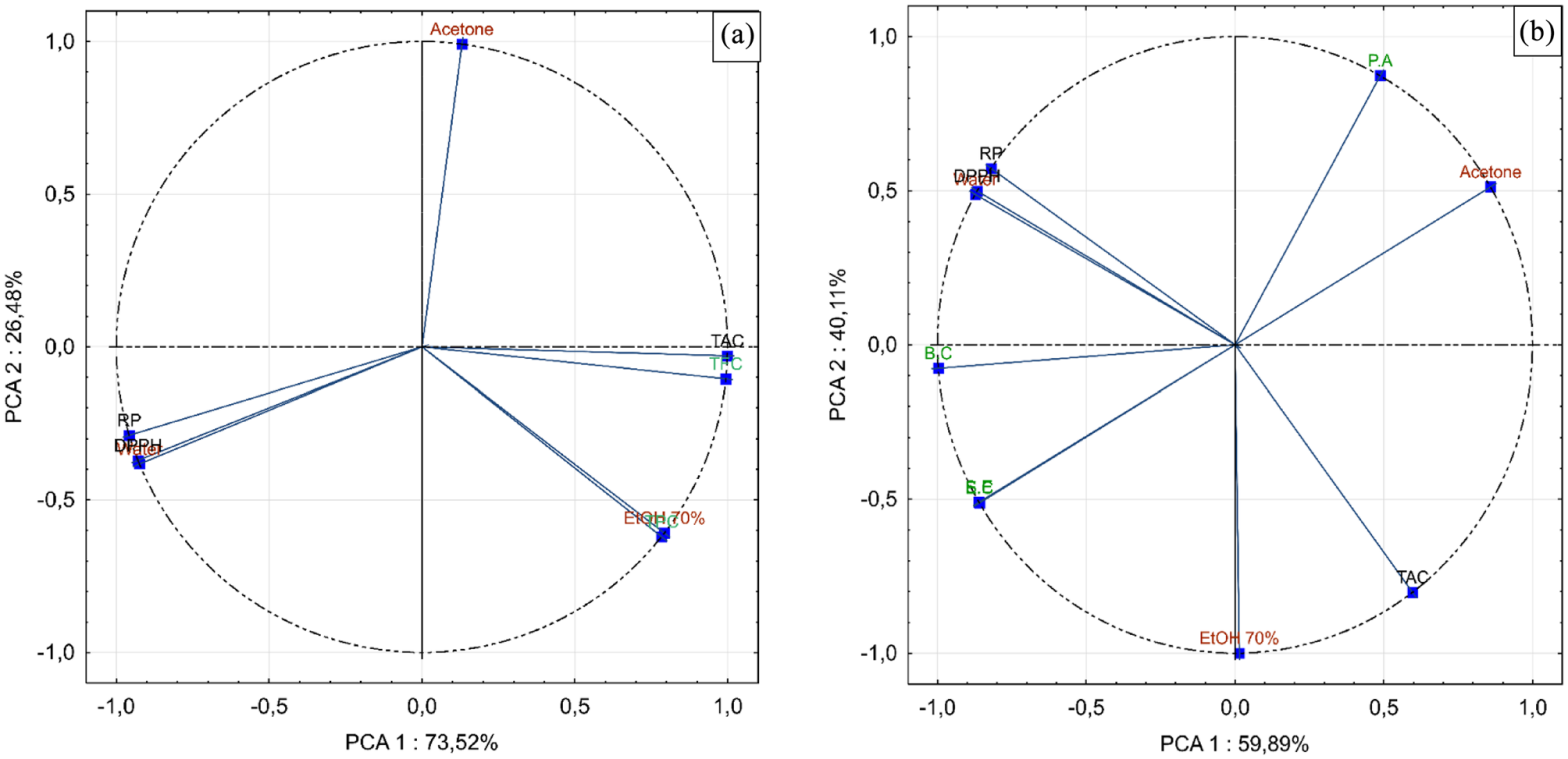
Principal component analysis (PCA). (**a**) Biplots for phenolic compounds and antioxidant activities of studied solvent extracts, (**b**) Biplots for antioxidant, and antibacterial activities of studied solvent extracts.

The previously acquired results were treated using the principal component analysis (PCA), and the key outputs are displayed in Figs. 6 (a, b). The principal component analysis (PCA) for total phenolic, flavonoid contents, and antioxidant activity of the studied extracts is shown in Fig. 6 (a). Most importantly, PC1 and PC2 explained 100% of the variation, which was sufficient to represent all of the selected variables. The PC1 against PC2 score plot indicates a significantly positive relationship between TPC, TFC, and TAC, demonstrating that the extracts with the highest potential for antioxidants also had the highest levels of TPC and TFC. These three parameters, particularly TPC, were shown to be positively correlated to the hydroethanolic solvent used in the extraction and negatively correlated to water. Moreover, DPPH and RP assays are regrouped negatively with the total phenolic and flavonoid contents, which supports the contribution of phenolic compounds in the DPPH scavenging ability and reducing the power of the different tested extracts, and the observed anti-*Bacillus cereus*, *Staphylococcus aureus* effects. While its negative part represents RP and DPPH.

Figure 6 (b) illustrates the PCA for antibacterial, and antioxidant activities of investigated extracts, with the two main components (PC1 and PC2) explaining 100% of the variation. The first component explained 59.81% of the variation and included TAC *and Pseudomonas aeruginosa* in its positive part, while the negative part included RP, DPPH, *Bacillus cereus*, and *Staphylococcus aureus*. Although the second component explained 40.11% and included water, RP, and DPPH in its positive part.

## Conclusion

The outcomes of this investigation demonstrated that the different *Mentha longifolia* leaf extracts contain a significant concentration of polyphenols and flavonoids. In addition, the phenolic composition of various polarities extracts indicated that the best yields of extracts with an ETOH of 70% were also the ones with the greatest variety of phenolic components. The investigated extracts demonstrated powerful antioxidant potential, with the highest level of activity shown by the hydroethanolic extract, followed by the acetonic and aqueous extracts. The antibacterial assays showed that all tested extracts demonstrated a powerful toxic effect against all tested bacterial strains, with a better effect of the acetonic extract against Gram-positive bacterial strains. Our findings suggest that the demonstrated properties of *Mentha longifolia* extracts can be used in various applications, including the development of a new antibiotic in the pharmaceutical sector and the preservation of fresh nutrition in the food industry. However, further in vivo experimental investigations are needed to evaluate the safety profile and eventual toxicity of Moroccan *Mentha longifolia* leaf extracts.

## Plant collection approval

No approval is needed to collect *Mentha longifolia* in Morocco for research purposes.

## IUCN Policy statement

The collection of plant material complies with relevant institutional, national, and international guidelines and legislation.

## Data Availability

All data generated or analyzed during this study are included in this published article.
